# GeneXpert and Community Health Workers Supported Patient Tracing for Tuberculosis Diagnosis in Conflict-Affected Border Areas in India

**DOI:** 10.3390/tropicalmed5010001

**Published:** 2019-12-21

**Authors:** Mrinalini Das, Dileep Pasupuleti, Srinivasa Rao, Stacy Sloan, Homa Mansoor, Stobdan Kalon, Farah Naz Hossain, Gabriella Ferlazzo, Petros Isaakidis

**Affiliations:** 1 Médecins Sans Frontières/Doctors Without Borders, Delhi 110024, India; msfocb-delhi-med@brussels.msf.org (H.M.); Msfocb-India-ops-strategic-advisor@brussels.msf.org (S.K.); msfocb-delhi-medco@brussels.msf.org (F.N.H.); 2 Médecins Sans Frontières/Doctors Without Borders, Bhadrachalam, Telangana 507111, India; 1drdileep1@gmail.com (D.P.); MSFOCB-Bhadrachalam-PMR@brussels.msf.org (S.S.); 3 District TB Office, RNTCP, Bhadrachalam district hospital, Bhadrachalam, Telangana 507111, India; golla.srinivasulu99@gmail.com; 4 Southern Africa Medical Unit, Médecins Sans Frontières, Cape Town 7925, South Africa; Gabriella.FERLAZZO@joburg.msf.org (G.F.); Petros.Isaakidis@joburg.msf.org (P.I.)

**Keywords:** sputum, health promotion, operational research, indigenous population

## Abstract

Médecins Sans Frontières (MSF) has been providing diagnosis and treatment for patients with tuberculosis (TB) via mobile clinics in conflict-affected border areas of Chhattisgarh, India since 2009. The study objectives were to determine the proportion of patients diagnosed with TB and those who were lost-to-follow-up (LTFU) prior to treatment initiation among patients with presumptive TB between April 2015 and August 2018. The study also compared bacteriological confirmation and pretreatment LTFU during two time periods: a) April 2015–August 2016 and b) April 2017–August 2018 (before and after the introduction of GeneXpert as a first diagnostic test). Community health workers (CHW) supported patient tracing. This study was a retrospective analysis of routine program data. Among 1042 patients with presumptive TB, 376 (36%) were diagnosed with TB. Of presumptive TB patients, the pretreatment LTFU was 7%. Upon comparing the two time-periods, bacteriological confirmation increased from 20% to 33%, while pretreatment LTFU decreased from 11% to 4%. TB diagnosis with GeneXpert as the first diagnostic test and CHW-supported patient tracing in a mobile-clinic model of care shows feasibility for replication in similar conflict-affected, hard to reach areas.

## 1. Introduction 

The management of tuberculosis (TB) is challenging for patients residing in remote and inaccessible areas. The scale of the challenge escalates when inaccessibility to healthcare increases due to conflict. Patients in these hard-to-reach areas need special attention from TB programmes and implementing partners [1].

India is a high-burden TB country, contributing to approximately a quarter of global incident TB cases. In 2018, the estimated number of TB cases in the country was 2,790,000 [2]. The border areas of central India (including four states, i.e., Chhattisgarh, Odisha, Telangana, Andhra Pradesh) have been affected by a long-standing, low-intensity, chronic conflict [3]. The majority of the population residing in these areas belong to various tribes and have limited access to healthcare services, including access to TB diagnosis and treatment facilities [4]. 

The Revised National TB Control Programme (RNTCP) has been providing TB care to remote and tribal populations [5]; however, these services in conflict-affected areas are often interrupted due to frequent instances of minor clashes. Basic healthcare services are provided at selected primary healthcare centers, but patients need to travel more than 50–100 km to access tertiary care services in district hospitals. 

Médecins Sans Frontières (MSF), a nongovernmental, medical humanitarian organization, has been providing primary healthcare services, including diagnosis and treatment for patients with TB, via mobile clinics in the chronic conflict-affected border areas of Chhattisgarh, India since 2009 [6]. A unique model of care in collaboration with RNTCP has been implemented, aiming at offering improved delivery of TB diagnosis and treatment services. GeneXpert in the nearby government hospital (Bhadrachalam district hospital) has been utilized as the first diagnostic test for TB diagnosis since January 2017. Community Health Workers (CHW) are trained in patient tracing (that is, in the follow up of patients) in order to minimize pretreatment loss-to-follow-up (LTFU). 

To date, there has been no documentation of this TB model of care in India. The aim of this study is to contribute to the body of evidence related to TB diagnostic delivery in conflict-affected and tribal areas, and to help policy makers and implementers to develop tailor-made, diagnostic strategies for such conflict-affected, hard-to-reach populations. 

The specific objectives of the study included determining the number and proportions of (1) patients diagnosed with TB, (2) pretreatment lost-to-follow-up patients (from first presentation for diagnosis up to the date of receipt of TB diagnosis results, and (3) to compare the proportion of bacteriological confirmation and pretreatment LTFU between two time periods: (a) April 2015–August 2016 and (b) April 2017 August 2018 (before and during the utilization of GeneXpert as a first diagnostic test for TB diagnosis). 

## 2. Materials and Methods

### 2.1. Study Design

This was a retrospective analysis of routinely collected clinical and programmatic data.

### 2.2. Setting

The state of Chhattisgarh in central India has a population of 26 million [7], including conflict-affected zones in the Sukma, Dantewada, and Bastar districs. Accurate information on the populations residing in the conflict-affected zones is not available [8]. The total number of notified TB cases in the Sukma district (where MSF TB Programme is operational) was 335 in 2018 [2].

#### TB Model of Care Description

MSF has been providing routine primary healthcare services, including TB care in the conflict-affected border areas with an estimated population of 90,000 since 2009 [6,9,10]. TB diagnosis and treatment is offered by a multidisciplinary team including doctors, nurses, counselors, health promoters, and CHWs. A doctor and nurse are TB focal points for the TB program. The nurses provide support with sample collections for TB diagnosis. The counselors provide information to patients and family members about TB signs/symptoms, treatment regimen, routes of TB transmission, and infection control. Health promoters carry out community sensitization sessions in villages every month on TB, malaria, diarrheal diseases, general hygiene, and sanitation. 

A group of local CHWs are trained the identify the symptoms/signs of TB and support the tracing of patients. In case patients miss appointments for two weeks or more, a CHW visits the patient at their residence in the villages. Repeated sensitization of CHWs (once every three months) is carried out by the TB focal points and health promoters. The CHWs are paid a fixed stipend every month. 

Since 2017, GeneXpert in the nearest district hospital in Bhadrachalam, Telangana has been utilized as the first diagnostic test for TB diagnosis in patients with presumptive TB. In India, studies have shown that GeneXpert has a sensitivity and specificity of 100% each for pulmonary TB samples and a sensitivity and specificity of 90.7% and 99.6% respectively for extra-pulmonary TB samples, in comparison with composite reference standards [11]. The patients with presumptive TB are requested to provide a spot sample in order to avoid the need to travel 10–15 kms to visit a clinic. Patients are given sputum containers for morning samples to be submitted on the next mobile clinic day (3 days later). The GeneXpert results (using spot samples) are given to patients on the next mobile van visit (the same visit when the morning sample is submitted); in cases whereby the GeneXpert result on the spot sample is negative, microscopy is performed on the morning sample. Patients are referred to the district hospital for a biopsy or chest X-ray, as required. For those with negative TB diagnostic results, a clinical decision is taken by the medical team. As laboratory results become available and are reported to the patient, pretreatment counseling is provided and treatment is initiated. 

### 2.3. Study Site and Population

All patients with presumptive TB who received care in the MSF TB Programme in border areas of Chhattisgarh between 01 April 2015 and 31 August 2018 were included. 

### 2.4. Data Variables and Sources, Data Analysis

The demographic (age, sex) and clinical characteristics (presence of cough, history of previous TB) of patients with presumptive TB, date of presentation, date of sputum collection, and diagnostic results (sputum, GeneXpert, Biopsy, Xray) were extracted from an electronic database and imported into STATA (version 11, StataCorp, College Station, Texas, USA) for analysis. TB diagnosis and pretreatment loss-to-follow up were summarized using frequency and proportions. Continuous variables such as age were summarized using median and inter-quartile range (IQR). Categorical variables (sex, previous history of TB, site of TB) were summarized as frequency and proportions. Associations between demographic and clinical characteristics and diagnosis of TB were assessed using a chi-square test and unadjusted relative risks (RR) with 95% Confidence Intervals (95% CI). A p value of less than 0.05 was considered statistically significant.

### 2.5. Operational Definitions

1. Presumptive TB: Presumptive TB refers to a patient who presented with symptoms or signs suggestive of TB [12]

2. Bacteriological confirmation: Presence of MTB+ in GeneXpert results; smear microscopy or culture evaluation was considered as a means of bacteriological confirmation

3. Clinically-diagnosed TB case: Patient diagnosed with active TB by a clinician on the basis of X-ray abnormalities and/or clinical evaluation. This includes smear-negative pulmonary TB and extra-pulmonary TB cases without laboratory confirmation.

4. Confirmed TB case: Patients with bacteriological confirmation or clinically-diagnosed TB 

5. Error: Failure to test for diagnosis of TB was termed as error, which included poor-quality of sputum, technical error of equipment, machine malfunction, etc.

6. Prediagnosis loss-to-follow-up: If the patients with presumptive TB, after the first consultation, did not visit the clinic to provide a sample for TB diagnosis, it was considered a prediagnosis loss-to-follow-up. 

7. Diagnosed TB loss-to-follow-up: If the patients were diagnosed with TB but did not visit the clinic for receipt of results within 1 month of THE initial consultation date, it was termed as diagnosed TB loss-to-follow-up.

8. Pretreatment loss-to-follow-up: The prediagnosis loss-to-follow-up and diagnosed TB loss-to-follow-up patients were together termed as pretreatment loss-to-follow-up. 

### 2.6. Ethics

This research fulfilled the exemption criteria set by the Médecins Sans Frontières Ethics Review Board for a posteriori analyses of routinely-collected clinical data. and thus, did not require MSF ERB review. It was conducted with permission from Medical Director, Operational Centre Brussels, Médecins Sans Frontières. Since it is a record-based study, we obtained a waiver from obtaining informed consent. Permission for conducting the study was sought from the National TB Programme of India (RNTCP).

## 3. Results

Among 1042 patients with presumptive TB identified in the program during April 2015 to August 2018, 376 (36%) were diagnosed with TB. The demographic and clinical characteristics of the patients with presumptive TB and those diagnosed with TB are shown in Table 1. The proportion of patients diagnosed with TB was largest (44.8%) in children aged 0–14 years compared to other age groups; it was similar in males (37.7%, 216/573) and females (34.1%, 160/469). Diagnosis of Pulmonary TB (PTB) was much more common than extra-pulmonary TB (82.9% versus 17.1%); however, a larger proportion of extra-pulmonary presumptive TB patients had confirmation of TB diagnosis than pulmonary TB patients (60.0% versus 32.4%). The younger age group [(0–14 years RR (95% CI): 1.5 (1.2–1.9); 15–24 years: 1.4 (1.1–1.8)] and extra-pulmonary TB [1.9 (1.6–2.1)] had a higher risk of developing TB. 

A total of 60 (5.8%) out of 1042 patients with presumptive TB were prediagnosis LTFU, while one died before providing sample for TB diagnosis. Of those who were diagnosed with TB (with bacteriological or clinical confirmation, n = 376), nine (2.4%) did not come back to receive the test results (termed as ‘diagnosed TB LTFU’) and did not initiate treatment during the study period. Thus, 69 (6.6%) of 1042 patients were pretreatment LTFU. Of those for whom a diagnosis of TB was made, 217 (57.7%), 49 (13%), and 110 (29.2%) had sputum-positive pulmonary TB, sputum-negative pulmonary TB, and extra-pulmonary TB, respectively (nontabulated). 

**Figure 1:**
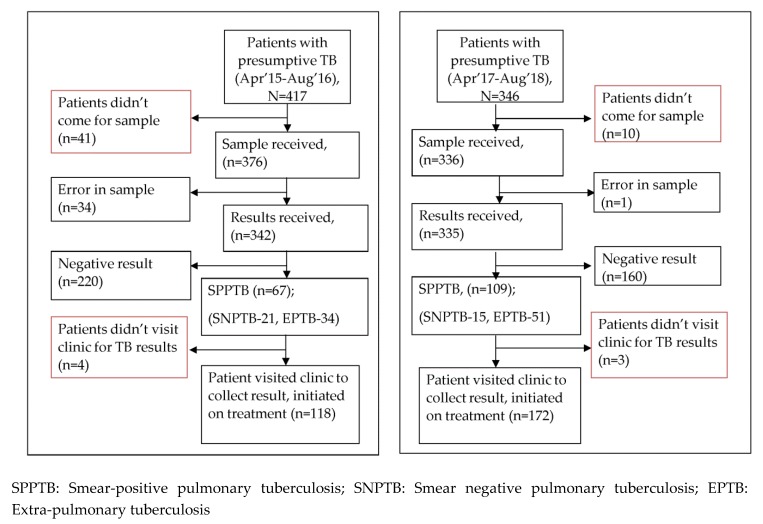
Bacteriological confirmation and pretreatment loss-to-follow-up during two time periods: (1) Apr. 2015–Aug. 2016 (Before GeneXpert was used as first diagnostic tool for TB-diagnosis), (2) Apr. 2017–Aug. 2018 (GeneXpert used for TB-diagnosis) in conflict-affected border areas in India.

Upon comparing the “before–after” time periods (Figure 1), bacteriological confirmation increased from 20% (67/342) to 33% (109/335). Errors in TB diagnoses decreased from 9% (34/376) to 0.1% (1/336). The pretreatment LTFU decreased from 11% (45/417) to 4% (13/346) during the study period.

## 4. Discussion

A model for TB diagnosis with the use of GeneXpert as a first test and CHW-supported patient tracing resulted in a 66% reduction of pretreatment loss-to-follow-up during 2015–2018 in a conflict-affected tribal area in India. 

More than one-third of patients identified with presumptive TB were diagnosed with active TB; this is higher than other studies reported in similar tribal areas (6.5% in the Bharia tribe of Madhya Pradesh, 12.5% in Maharashtra, and 21% in the Sahariya tribe of central India) [13,14,15]. This could be due to the availability of a trained medical team for supporting TB diagnosis. Further, the availability of GeneXpert as a first diagnostic test for presumptive TB patients in government district hospitals likely contributed to the increased number of detected cases [16,17], some of which may have been missed earlier. Investments for the continuous operation of GeneXpert must be considered by TB programs. The availability and accessibility of diagnostic tools like GeneXpert [17] and the implementation by a trained team [18] help in early and appropriate TB diagnoses in these hard-to-reach areas. 

Studies in conflict areas across the globe have reported multiple challenges of access to healthcare services [19,20,21]. Few TB programs have been successful in delivering treatment in conflict areas by adapting to local needs [22]. Other than direct medical care under the National TB Programme, intersectoral measures such as access to a public distribution system, nutritional support, social welfare schemes, and security measures at the central and state levels will help to minimize the TB burden. 

The proportion of children and young adults was low in this group of patients with presumptive TB; however, the proportion of diagnosed TB was high compared to other age groups. As children are considered proxy indicators of TB transmission [23], it may be noted that the burden of TB is high in this tribal population, as it is in other tribal populations in the country [15]. The national TB program in country, with the involvement of other NGOs and stakeholders, must devise tailored approaches for the provision of improved diagnoses and treatment in children [24,25]. 

The proportion of diagnosed EPTB cases among presumptive EPTB cases was higher than expected. This may hint at the late arrival of EPTB cases for diagnosis [26] and lower awareness about extrapulmonary signs of TB among the community [27]. EPTB is often considered low priority by TB programs, as it does not lead to the transmission of infection [28]. Strategies must be proposed to improve access to diagnostics for EPTB (fine needle aspiration cytology, biopsy) in the nearest healthcare facilities [29].

The proportion of pretreatment loss-to-follow-up is lower than in other studies in the country [16,30]. This could be due to the dedicated and trained CHW personnel who were responsible for tracing the patients, in case they missed mobile clinic appointments. The CHW were from the same communities, and therefore, were likely to be more accepted by the population [31,32]. However, the uncertain security situation and the movements among the population to nearby cities during nonharvest seasons (for work) posed major challenges in tracing patients.

The study had several limitations. The mobile clinics often faced limitations in routine activities in case of security issues. It was difficult for CHWs to trace patients from far off villages, deep in forest areas. The study results may not be generalized to other tribal areas, as the study is based on a resource-intensive TB program of a medical humanitarian NGO, working in the same area for about a decade. Most of the authors of the study were employees of the NGO, though the research team was not involved in the implementation of the program in the field. We believe that this might have moderated the potential bias in reporting on the implementation of the program. Despite the limitations, this is one of the first studies from a conflict-affected, tribal area in India, describing a unique diagnostic TB model of care based on a routine, mobile clinic-based TB program of an NGO, and thereby documenting the reality on the ground.

## 5. Conclusions 

A mobile-clinic model of care for TB shows feasibility for replication in similar ‘hard to reach’ (namely conflict-affected and tribal) areas for improved access to quality TB diagnosis and care. Improved diagnostics such GeneXpert and utilizing CHWs from the communities for tracing and following the patients would be beneficial for early TB diagnoses.

## Figures and Tables

**Table 1:** Demographic and clinical characteristics of patients with presumptive TB and those diagnosed with TB in conflict-affected border areas in India, 2015–2018.

Characteristic	Patients with Presumptive TB * n (%)	Patients Diagnosed with TB ** n (%)	Unadjusted RR (95% CI)	Chi-Square (*p*-Value)
**Total**	**1042**	**376 (36.1)**		
Age group (years) (N = 1038)				
0–14	134 (12.9)	60 (44.8)	1.5 (**1.2–1.9**)	12.01 (**0.02**)
15–24	117 (11.2)	49 (41.9)	1.4 (**1.1–1.8**)	
25–34	197 (19.0)	75 (38.1)	1.3 (0.9–1.6)	
35–44	231 (22.3)	84 (36.4)	1.2 (0.9–1.5)	
45 and above	359 (34.6)	108 (30.1)	1	
Sex				
Male	573 (55.0)	216 (37.7)	1.1 (0.9–1.3)	1.43 (0.23)
Female	469 (45.0)	160 (34.1)	1	
TB site (N = 997)				
Pulmonary	827 (82.9)	268 (32.4)	1	46.0 (**<0.01**)
Extra-pulmonary	170 (17.1)	102 (60.0)	1.9 (**1.6–2.1**)	
Previous TB (N = 995)				
Yes	201 (20.2)	75 (37.3)	1.0 (0.8–1.2)	0.01 (0.9)
No	794 (79.8)	293 (36.9)	1	

* Column percentage, ** Row percentages, Unadjusted RR: Unadjusted Relative Risk; CI: Confidence Intervals.
